# Development and Evaluation of a New Serious Game for Continuing Medical Education of General Practitioners (Hygie): Double-Blinded Randomized Controlled Trial

**DOI:** 10.2196/12669

**Published:** 2019-11-20

**Authors:** Louis-Baptiste Jaunay, Philippe Zerr, Lino Peguin, Léandre Renouard, Anne-Sophie Ivanoff, Hervé Picard, James Griffith, Olivier Chassany, Martin Duracinsky

**Affiliations:** 1 Département de Médecine Générale Sorbonne Paris Cité Université Paris-Descartes Paris France; 2 Département de Médecine Générale Sorbonne Paris Cité Université Paris-Diderot Paris France; 3 Service de Recherche Clinique Fondation Rothschild Paris France; 4 Pole Recherche et Evaluation Scientifique Cabinet Ipso Paris France; 5 Department of Medical Social Sciences Feinberg School of Medicine Northwestern University Chicago, IL United States; 6 Patient-Reported Outcomes Research Sorbonne Paris Cité Université Paris-Diderot Paris France; 7 Unité de Recherche Clinique en Economie de la Santé Hôpital Hôtel-Dieu Assistance Publique Hôpital de Paris Paris France; 8 Médecine Interne et Immunologie Clinique Hôpital Bicêtre Assistance Publique Hôpital de Paris Paris France

**Keywords:** general practice, continuing medical education, evidence-based medicine, video games, randomized controlled trial, pedagogy

## Abstract

**Background:**

Continuing medical education is important but time-consuming for general practitioners (GPs). Current learning approaches are limited and lack the ability to engage some practitioners. Serious games are new learning approaches that use video games as engaging teaching material. They have significant advantages in terms of efficiency and dissemination.

**Objective:**

The aim of this study was to create a serious game and to evaluate it in terms of effectiveness and satisfaction, comparing it with a traditional method of continuing education—article reading.

**Methods:**

We produced a prototype video game called *Hygie* on the 5 most common reasons of consultation in general practice using 9 articles from independent evidence-based medicine journals (reviews from *Prescrire* and *Minerva*). We created 51 clinical cases. We then conducted a double-blinded randomized trial comparing the learning provided by a week of access to the game versus source articles. Participants were GPs involved as resident supervisors in 14 French university departments of family practice, recruited by email. Primary outcomes were (1) mean final knowledge score completed 3 to 5 weeks after the end of the intervention and (2) mean difference between knowledge pretest (before intervention) and posttest (3 to 5 weeks after intervention) scores, both scaled on 10 points. Secondary outcomes were transfer of knowledge learned to practice, satisfaction, and time spent playing.

**Results:**

A total of 269 GPs agreed to participate in the study. Characteristics of participants were similar between learning groups. There was no difference between groups on the mean score of the final knowledge test, with scores of 4.9 (95% CI 4.6-5.2) in the *Hygie* group and 4.6 (95% CI 4.2-4.9) in the reading group (*P*=.21). There was a mean difference score between knowledge pre- and posttests, with significantly superior performance for *Hygie* (mean gain of 1.6 in the *Hygie* group and 0.9 in the reading group; *P*=.02), demonstrating a more efficient and persistent learning with Hygie. The rate of participants that reported to have used the knowledge they learned through the teaching material was significantly superior in the *Hygie* group: 77% (47/61) in the *Hygie* group and 53% (25/47) in the reading group; odds ratio 2.9, 95% CI 1.2-7.4. Moreover, 87% of the opinions were favorable, indicating that *Hygie* is of interest for updating medical knowledge. Qualitative data showed that learners enjoyed *Hygie* especially for its playful, interactive, and stimulating aspects.

**Conclusions:**

We conclude that *Hygie* can diversify the offering for continuing education for GPs in an effective, pleasant, and evidence-based way.

**Trial Registration:**

ClinicalTrials.gov NCT03486275; https://clinicaltrials.gov/ct2/show/NCT03486275

## Introduction

### Background

General practitioners (GPs) update their medical knowledge throughout their professional life to maintain knowledge acquired during their initial studies and to be abreast of the latest scientific advances.

Continuing medical education, however, can be tedious and sporadic because a considerable amount of new medical data and new literature are being continuously released, varying in quality and accessibility. The busy practitioner has limited time to consult this information [1-3], and traditional teaching methods such as lectures and group discussion have small and short-lasting effects [4]. As a result, clinical care may not be in line with the latest science, leading to poorer health outcomes [5]. Thus, new, efficient, and stimulating teaching methods are required.

New teaching materials called *serious games* are efficient [6,7] and easily disseminated methods for education [8]. Indeed, they offer the possibility of combining learning activities such as testing [9], feedback [10], spaced repetition [11], and problem-based learning [12,13] with a positive experience. Learning challenges can be provided by these games [14,15] in a risk-free environment [16]. Therefore, serious games give active participation and autonomy to the learner, both of which are crucial qualities in adult education [17].

Few serious games have been developed with the goal of facilitating continuous medical education for health professionals [18] and GPs [19,20]. To our knowledge, no existing game covers several topics related to family medicine.

### Objectives

The aim of this study was to develop a prototype of a new serious game called *Hygie* for continuing medical education for the GP and to assess its effectiveness and user acceptance as compared with a traditional activity (article reading) in a randomized trial.

## Methods

### Design and Development of Hygie

We produced a prototype video game called *Hygie* in which the player is a GP in the process of treating several patients. We defined topics for this prototype based on the 5 most frequent reasons for consultation in France [21]: hypertension, health check and prevention, dyslipidemia, acute fever, and rhinopharyngitis.

For these 5 topics, we reviewed 9 articles in 2 French evidence-based journals: 6 from *Prescrire* and 3 from *Minerva* [22-30]. We selected these 2 journals because they provide robust evidence-based recommendations and are strictly independent from industrial and institutional influences.

From these 9 articles, we created 51 short clinical cases, each having 1 question that could be answered either by multiple choice or free text.

**Figure 1:**
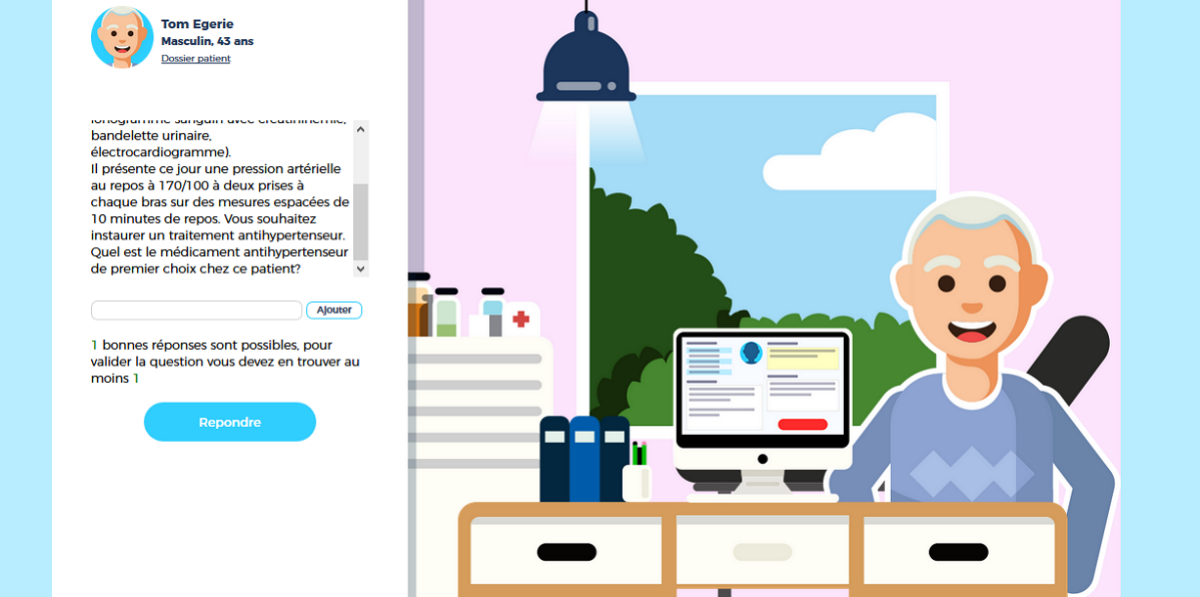
Start of a clinical case in *Hygie*.

The game was coded using HTML 5, Cascading Style Sheets 3, JavaScript (ECMAScript 2015), and Hypertext Preprocessor (PHP) 7. Graphics were created using Adobe Illustrator and Adobe Photoshop (Figure 1).

Learning methods incorporated into the game included statement of educational objectives, immersion in a general medical consultation setting, problem-based learning with active restitution of knowledge, spaced recall, stimulation of intrinsic motivation by earning points, and having goals and levels with a “final boss” for each level. Humoristic elements such as puns in patients’ names were included to maximize engagement.

A preliminary test phase was conducted with 11 GPs and 9 residents in general practice. The preliminary test allowed us to detect and solve bugs, clarify questions, and sort questions into 5 levels of difficulty.

The prototype of the game is freely accessible on the Web [31].

### Study Design

We performed a double-blind randomized controlled trial to assess the effectiveness of *Hygie* as a method for continuing education for GPs as compared with a traditional article reading activity with the same content.

We asked all 35 French university departments of family practice to contact GPs involved as resident supervisors by email to participate in a real-life experience learning where they would have access to an electronic learning (e-learning) teaching material for 7 days, without mentioning the nature of the teaching materials. Institutional affiliations of the investigators were indicated at the end of the email.

Information was delivered to participants about the purpose, the duration, the time to devote to the study, and anonymization of results.

After agreeing to participate, GPs accessed the study website where they completed a demographic questionnaire, a knowledge pretest of 5 questions on each of the 5 reasons for consultation. They were randomized using the rand function of PHP language (allocation ratio 1:1) to either the intervention group (Web access to *Hygie* for 1 week) or to the control group (access online to the 9 articles). Participants had an individual login, allowing them to access only the teaching material assigned to them. They did not know if they were assigned to the intervention or control group and did not know which intervention was performed in the other group. Data were collected on a Structured Query Language database.

After 1 week of free access to their respective teaching material (serious game *Hygie* vs articles), access was terminated. Reminders were sent to the 2 groups within 3 and 6 days of access to teaching material.

**Figure 2:**
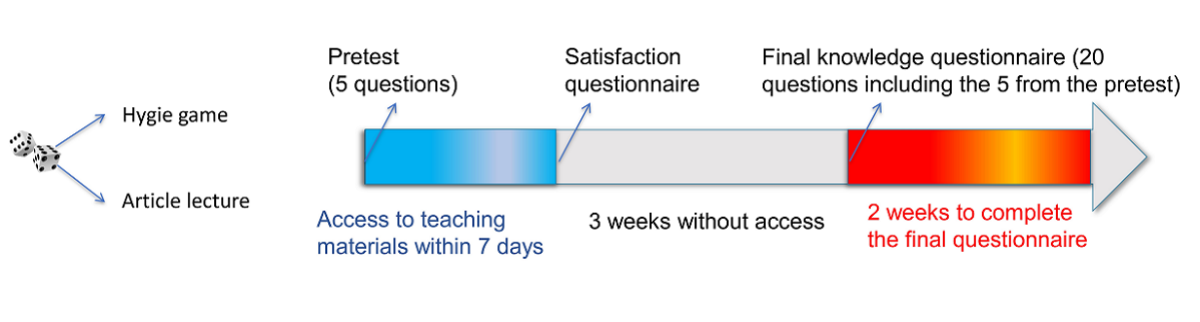
Study design.

After 3 weeks without access to the teaching materials, participants received a final, 20-item knowledge questionnaire (Figure 2). Among the 20 questions, 5 were common with pretest. Only those participants who had completed the final questionnaire were analyzed.

Primary outcomes were (1) dynamic and (2) static knowledge assessed by questionnaires:

1. A Dynamic Questionnaire-5 (DQ-5) with 5 items compared individual change of score between pretest (before intervention) and posttest (3-5 weeks after intervention). It was a 5-item questionnaire with each of the 5 questions weighted by a scale ranging from 1 to 3 according to its importance for practice and a global score from 0 to 14. The goal of this questionnaire was to assess progression of each participants. For a simpler interpretation, we scaled the DQ-5 score to be out of 10 rather than 14.
2. A Static Questionnaire-20 (SQ-20) measured mean final score 3 to 5 weeks after intervention. It was a 20-item questionnaire (5 of the dynamic questionnaire plus 15 other questions), with each of the 20 questions weighted by a scale ranging from 1 to 3 according to its importance for practice and a global score from 0 to 58. The goal of this questionnaire was to compare groups, minimizing the potential carryover effects induced by the pretest questionnaire. Like for the DQ-5, for interpretation, we rescaled the SQ-20 to a 0 to 10 scale (rather than 0-58).

Here is an example of a knowledge question that appears in both dynamic and static questionnaires and the scoring method:

Question: Which cholesterol-lowering drugs have shown a decrease in mortality and morbidity?

Expected answers (free text): pravastatin, simvastatin.

Scoring method: It was rated 3 points out of 14;

If the 2 right molecules (pravastatin and simvastatin) are mentioned: 3 points

If 1 good molecule among pravastatin and simvastatin is mentioned: 1 point

In all other cases: 0 points.

The 2 knowledge questionnaires and their scale were written from the source articles by 3 experienced physicians who had no information about the game content, with instructions to identify practice-relevant issues in the articles. Participants’ questionnaires were scored blindly by a physician not involved in the other stages of the study.

Secondary outcomes were (1) the use in medical practice of the knowledge acquired through the teaching material assessed at the time of the final questionnaire (participants answered the question “In the course of your practice, did you use the knowledge you learned through the teaching material?”), (2) time spent playing by participants assigned to *Hygie*, and (3) a satisfaction questionnaire. The satisfaction questionnaire, composed of 8 questions and completed at the end of the 1-week learning period, included quantitative and qualitative data about participant satisfaction, time reported as spent on the materials, and additional demographic data (eg, workplace and usual training materials for continuing education). Qualitative data were analyzed by content for themes related to effective learning as well as to illuminate potential strengths and weaknesses of *Hygie*. The average total time spent on the *Hygie* game was measured via server usage data. Average total time spent on the articles was not collected because participants could download the articles and read it offline.

### Statistical Analysis

The answers to the knowledge and satisfaction questionnaires were collected on the framaform website. Statistical analyses were performed using R software (R Foundation for Statistical Computing) [32]. The 2 groups were compared using Fisher exact tests for nominal variables and Welch *t* tests for quantitative variables. Differences with *P*<.05 were considered significant.

### Sample Size

The number of participants required with 80% power (1−beta) and 5% type I error was estimated before the study. A total of 128 participants were needed to detect a difference of 2 points out of 10 between the groups on the final questionnaire, assuming that the participants in the *Hygie* group had a final score of 8 out of 10 on average.

### Ethics

Participation was anonymous and voluntary. Participants began the study by clicking a link to teaching materials.

The study was approved by the Committee for the Evaluation of the Ethics of Research Projects of hospital Robert Debré n° 2017/359.

## Results

### Participant Statistics

A total of 14 university departments from 8 French regions accepted to participate in this study. A total of 3398 GPs were invited to participate in this study by email. Of these, 269 participants (7.9%) accepted to participate in the study. Recruitment occurred between May 31, 2017, and June 27, 2017. A total of 108 participants completed the study and were analyzed. There was no difference of baseline characteristics between participants who completed and participants who did not complete the study.

**Figure 3:**
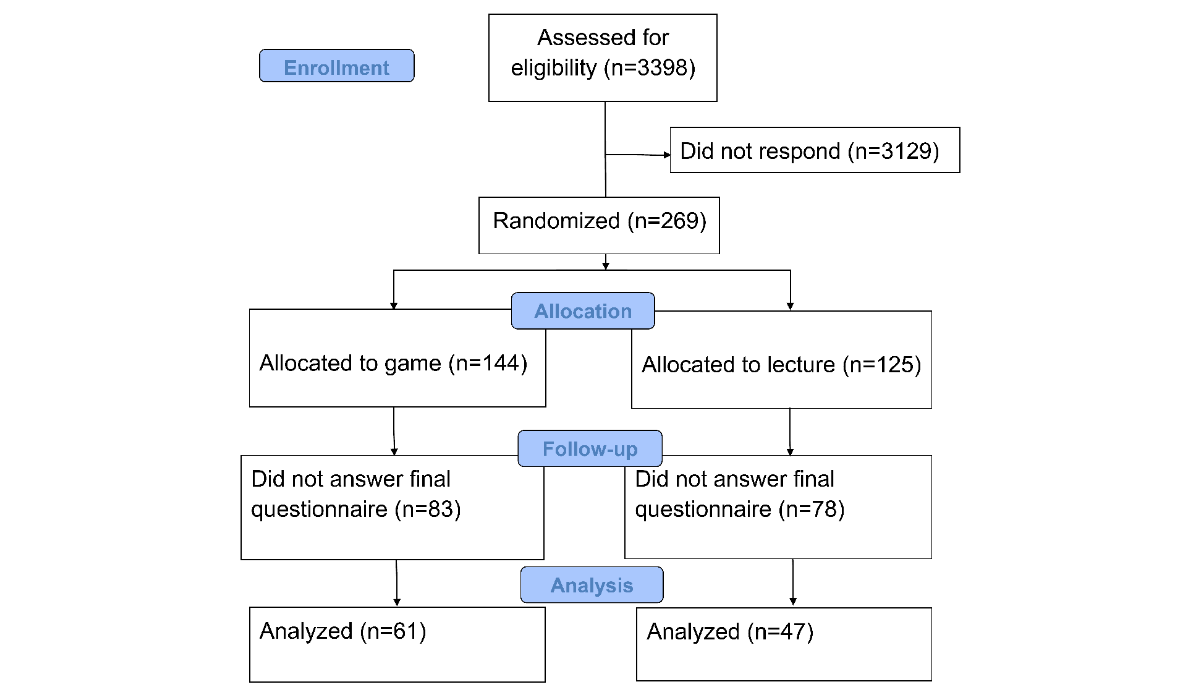
Flow diagram.

The inclusion flow diagram according to Consolidated Standards of Reporting Trials recommendations [33] is shown in Figure 3.

Baseline characteristics of participants in both groups were comparable (Table 1). Mean age was 40.9 years, there was a majority of women, and an urban setting was the most common. The most widely used continuing education method was reading print journals.

The DQ-5 pretest mean score was identical in the 2 groups: 3.4 (95% CI 2.9-3.8) in the intervention group and 3.8 (95% CI 3.2-4.3) in the control group (*P*=.27, not significant).

Average time between stopping access to support and completing the final questionnaire was similar in the 2 groups: 25.3 days (95% CI 24.2-26.5) in the *Hygie* group and 27.5 days (95% CI 26.3-28.7) in the control group.

**Table 1:** Characteristics of analyzed participants allocated to each intervention (*Hygie* or control) at baseline (n=108).

Characteristics of participants		*Hygie* group (n=61)	Reading group (n=47)
**Gender, n (%)**			
	Female	30 (49)	27 (57)
	Male	31 (51)	20 (43)
Mean age (min-max)		39.8 (27-67)	42.4 (28-64)
Dynamic Questionnaire-5 pretest, mean score (95% CI)		3.4 (2.9-3.8)	3.8 (3.2-4.3)
**Workplace setting, n (%)**			
	Rural	31 (51)	35 (74)
	Semirural	23 (38)	7 (15)
	Urban	7 (11)	6 (13)
**Continuous teaching material, n (%)**			
	Paper journals	51 (84)	39 (83)
	Internet journals	23 (38)	23 (49)
	Internet sites	36 (59)	33 (70)
	Onsite courses	48 (79)	33 (70)
	Peer group training	35 (57)	20 (43)
	Medical visitors	5	12

### Outcomes

#### Knowledge

The final SQ-20 mean score was similar in the 2 groups: *Hygie* group 4.9 (95% CI 4.6-5.2) and control group 4.6 (95% CI 4.2-4.9; *P*=.21, not significant).

The final DQ-5 mean score (5-item posttest) was also similar in the 2 groups: *Hygie* group 5.0 (95% CI 4.6-5.4) and control group 4.7 (95% CI 4.2-5.1; *P*=.26, not significant).

The mean individual change of DQ-5 score between pre- and posttest was significantly superior to 0 in the *Hygie* group with a mean gain of 1.6 (95% CI 1.2-2.1; *P*<.001) and in control group with a mean gain of 0.9 (95% CI 0.5-1.4; *P*<.001).

**Figure 4:**
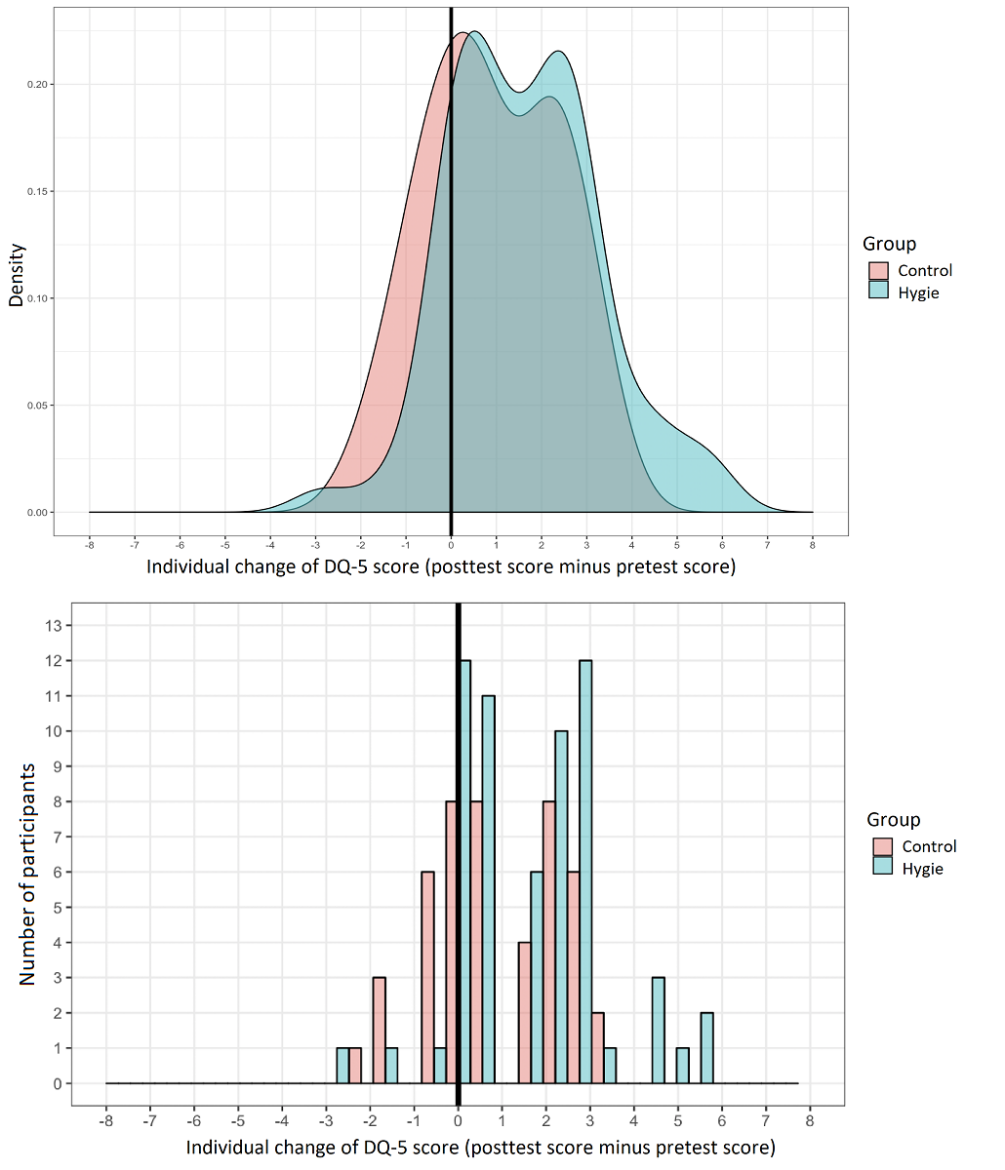
Individual change of score in both groups, shown as density and number of participants. DQ-5: Dynamic Questionnaire-5.

For the critical test of our trial, this mean individual change of DQ-5 score between pre- and posttest at 3 to 5 weeks was significantly superior in the *Hygie* group compared with the reading group, with a difference of 0.7 (95% CI 0.1-1.3; *P*=.02; Figure 4).

#### Transfer to practice

For the question “In the course of your practice, did you use the knowledge that you learned through the teaching material?,” the percentage of participants reporting “yes” was significantly greater in the *Hygie* group (77% in the *Hygie* group vs 53% in the reading group; odds ratio 2.9, 95% CI 1.2-7.4; Table 2).

**Table 2:** General practitioners’ responses to “In the course of your practice, did you use the knowledge you learned through the teaching material?”

Response	*Hygie* group, n (%)	Reading group, n (%)
Yes	47 (77)	25 (53)
No	14 (23)	22 (47)

Players could rate clinical cases in terms of “usefulness to practice” just after resolving the cases. A total of 1464 clinical case scores were given, and the average score given was 4.14 out of 5.

#### Satisfaction and Qualitative Data

In the satisfaction survey, 87% of *Hygie* group participants answered “yes” to the question “Do you think that *Hygie* is of interest for updating your medical knowledge?” and 75% answered “yes” to the question “Do you think that *Hygie* should be allowed for continuing education credits?”.

The qualitative reasons spontaneously mentioned by the participants also justified *Hygie*, including the following themes:

- Effective learning: the characteristics of the game (subthemes mentioned the following: speed, simple learning, and effective information assimilation), informative content (key messages, relevance of themes, clarity, and referenced responses), and its mechanisms (repetition of clinical cases promoting memorization, cognitive conflict that allows for better memorization, and allows one to learn test with real-life scenarios).
- An enjoyable experience (subthemes mentioned the following: playful and fun) with stimulating challenges (challenging stimulation and real-time style mimics the clinic): 36% of participants of the *Hygie* group answered “yes” to the question “Did this session make you want to consult medical journals more regularly or take out a subscription?,” which suggests that gaming encourages players to read journals, considering that 73% of GPs already reported consulting *Prescrire* regularly and 12% reported consulting Minerva.

#### Time Spent on Supports

The average total time spent on the *Hygie* game, measured via server usage data for the included participants, was 43 min. The average time per game session was 10 min and 50 seconds. Participants self-reported the time they spent on learning materials in the satisfaction questionnaire through a discontinuous quantitative variable. The most common responses were “45 to 60 minutes” in the *Hygie* group and “10 to 20 minutes” in the reading group.

#### Success Rate and Comments on Game Questions

The overall success rate for clinical cases was 67%. Participants’ comments on clinical cases reflected the cognitive conflict produced in players by the system of interaction between the GPs knowledge and the “model” proposed by the game: some agreed with the answer (eg, “bravo”), whereas others criticized the clarity of the question (eg, “one could specify [...]”) or criticized the answer based on their practice (eg, “I would have liked to do [...] before treating”) or other sources (eg, “recommendations on this topic include [...]”).

## Discussion

### Principal Findings

To our knowledge, *Hygie* is the first continuing education material of this type; it is the first educational video game developed for and by GPs. *Hygie* was created without external funding and independently of the pharmaceutical and medical device industries. Moreover, it is based on reliable sources that are helpful to GPs in maintaining and expending their knowledge. Finally, it is unique because of its extensive evaluation among a significant number of GPs from several regions of France. The use of both a double-blinded randomized trial and a satisfaction questionnaire evaluation differentiates *Hygie* from other serious health games in existence, with a few exceptions such as InsuOnline [20]. Our study shows that it is feasible to create an engaging educational video game, including validation in a randomized trial, without influence of public or private financing.

Our results have shown that giving access to the *Hygie* game to GPs in “real life” conditions (ie, where learner decided when, where, and how much time he or she wants to spend learning) results in a persistent learning at 3 to 5 weeks. Furthermore, giving access to *Hygie* resulted in a better improvement in medical knowledge compared with giving access to articles, which is the traditional method. In addition, this knowledge seems to be more easily transferable to medical practice, as shown by the greater proportion of GPs reporting having used the knowledge in their own practices as compared with traditional journal article reading. This result suggests that serious games may engender better transfer of knowledge to real-life situations by actively engaging the learner.

No significant difference was found on the final questionnaire score, which is consistent with a previous study [20] and may suggest that journal article reading can still lead to sufficient knowledge for continuing education but that *Hygie* is at least noninferior to traditional methods.

### Limitations and Strengths

There were some limitations to our study.

Recruitment was limited to GPs who were resident supervisors. This population is representative of the French GP population with some particularities such as a higher proportion of women, an underrepresented 45 to 54 years age group, a majority group practice, and a lower weekly working time [34]. Another bias is that participants were volunteered for the study after reading the email solicitation that offered to try a “new continuing education material.” Thus, it was possible that this population of GPs was especially interested in updating their medical knowledge; this is supported by the proportion of physicians declaring reading the *Prescrire* journal in our study (70%), which is much higher than the proportion of French GPs subscribing to *Prescrire* (18.1% of GPs subscribed to *Prescrire* in 2016) [35].

The GPs’ positive response rate for participating in the study was 7.9% (269 included out of 3398 requested), which is comparable with the average response rate in this population [36] but prevented us from reaching the number of participants suggested by our power analysis. The real response rate cannot be definitively known because it is possible that some emails failed to reach potential GPs and were not read.

Contamination bias between groups is a potential limitation, but limited access to 1 of the 2 teaching materials through the login and individual working environment of French GPs has limited this possibility.

More than half (60%) of the participants did not complete the study, which may have been a consequence of the “real life” conditions of our trial (unconstrained use) and the fact that the study took place during the summer holidays. The similar number of participants who did not complete the study in the 2 groups (58% in the *Hygie* group and 62% in the control group) suggests that the reasons for not participating are not related to the nature of the teaching material. Similarly, it can be assumed that the influence of reminders during the week of access to teaching materials, compared with routine use, was similar for both groups. However, the final sample size was smaller than the number calculated as required. A lack of power may explain that 1 of the 2 end points did not reach statistical significance.

Knowledge and satisfaction questionnaire have not been previously validated because they have been made to match the content of the teaching materials. The use of customized instruments is strongly recommended for the evaluation of serious games by Moreno-Ger [37], who argues that generic questionnaires are usually not useful for assessing games that can be very different in their objectives, target audiences, and needs. However, GPs experienced in medical pedagogy reviewed and improved these questionnaires, which was then pilot tested.

The scores obtained by the participants in the pretest knowledge questionnaire were surprisingly low. The lack of knowledge of clinical practice recommendations by French GPs is known in the literature [38]. In addition, the knowledge questionnaire presented several difficulties: free-text responses and needing to know recent evidence-based recommendations. The improvement in scores between pretest and posttest, although significant in both groups, may appear small. In addition to the difficulty of the questionnaire, which may have limited the progression of participants, this slight increase can be explained by the forgetting of knowledge.

The duration between the end of access to the teaching materials and the final test questionnaire was chosen at 3 to 5 weeks to evaluate long-term memorization, the most relevant type of memorization for the GP, and to limit the number of people lost to follow-up over a too long a period. We based our decision of follow-up period on a study conducted in 2008 evaluating the long-term memorization by residents of recommendations on type 2 diabetes learned via an internet tutorial [39]. Subjects were randomized into 6 groups that varied the time between the tutorial and the knowledge assessment: without delay and with delay of 1 day, 3 days, 8 days, 21 days, and 55 days. At 21 days, the interns had forgotten more than half of the knowledge learned compared with those assessed without any delay, suggesting that this duration allows long-term learning to be assessed.

The time spent on learning material could not be collected automatically in control group. These data would have provided an additional element of comparison between the 2 groups. However, as the groups were randomized, the effect of individual preferences regarding time allocation can be assumed to be balanced between groups. The self-reported time of participants was more than 2 times longer in the *Hygie* group; it is possible that this result indicates that *Hygie* is more time-consuming than reading. However, in the “real life” conditions of this trial, where each participant chose the time spent on the support, it seems that this result is rather in favor of *Hygie*’s interest. That is, this educational support seems particularly engaging in this population of GPs, who are known to lack time and motivation for continuing education.

### Conclusions

A very favorable reception was given by most GPs who used the *Hygie* game, particularly for its playful, interactive, and stimulating aspects, which supported the engaging learning experience.

In this study, many GPs spent much time on *Hygie*, commenting favorably on the clinical cases and the resulting learning experiences. A large proportion of participants expressed a desire to use it regularly for continuing education. In addition, *Hygie* serious game inspired many participants to subscribe to journals, which implies a synergy of this novel approach with the traditional article reading approach.

Our pragmatic study suggests that under usual conditions with e-learning teaching material, *Hygie* game can be an effective, pleasant, and engaging method for continuing education of GPs. It can be widely disseminated at low cost. Its modular content allows for future adaptation and improvement, and immersive qualities in a virtual reality where errors are not detrimental to patients render it an exciting next direction for adult learning among GPs and other physicians. In the future, we could evaluate the appropriation of this tool by GPs and their ability to improve it.

## Appendix

Multimedia Appendix 1 CONSORT-EHEALTH checklist (V 1.6.1).

## Abbreviations

DQ-5: Dynamic Questionnaire-5
e-learning: electronic learning
GP: general practitioner
PHP: Hypertext Preprocessor
SQ-20: Static Questionnaire-20
